# Supplementary material for: Direct Impact of the Air on Mutant Cells for Mutagenicity Assessments in Urban Environments
Source: Microorganisms. 2023 Dec 19;12(1):3. doi: 10.3390/microorganisms12010003 (PMC10820087; doi:10.3390/microorganisms12010003)
Supplement: Supplementary file 1 [file microorganisms-12-00003-s001.zip › microorganisms-2760931-supplementary.pdf]

Supplementary S1

<u>1st day:</u>

-The base media have been prepared for the contact plates (RodacTM Contact Plates, VWR, USA). The medium was autoclaved at 121 ° C for 20 minutes and the sterile Glucose (50ml/L) and Vogel-Bonner medium (20ml/L) were added.

- *Salmonella typhimurium* inoculum is prepared in Nutrient Broth (Oxoid N°29) in presence of ampicillin (2.15 µg/ml).

<u>2nd day:</u>

A second medium (soft agar) was prepared, based on Agar (0.6g/100ml) and NaCl (0.5g/100ml), to which, a Histidine-Biotin solution (1ml/100ml) and selected antibiotics were added. Starting from this solution, 2.5ml aliquots are prepared inside test tubes. Different substances are added to each aliquot of soft agar depending on the type of plate to be prepared (white, controls both negative and positive, and samples) as following showed. After all the substances inside the different tubes have been added, the soft agar obtained has been agitated, poured, and distributed on the contact plates.

- White: plates containing only the medium, to verify that this does not contain any contamination;
- Negative control: 100 µl respectively of the three *Salmonella typhimurium* strain inoculums (TA98, TA100, YG1024) with the aim of evaluating spontaneous reverse mutation;
- Positive controls: 100 µl of each strain (in different tubes), with the addition of a mutagenic substance (0.1 µg/plate) with a known spectrum of action (Nitrofluorene for strains TA98 and YG1024, and Sodium Azide for strain TA100), performed to evaluate the effectiveness of the test.
- Samples: 100 µl respectively of the three *Salmonella typhimurium* strain inoculums (TA98, TA100, YG1024) will included in different plates and then, at the monitoring station, exposed to the various volume of air (400 ,700, 1000L).

After all the substances inside the different tubes have been added, the soft agar obtained has been agitated, poured, and distributed on the contact plates.

All the plates were transported to the sampling station and come back into the lab avoiding confounding factor due to the transport. Al the plates were incubated for 48 hours at 42°C.

Figure S1

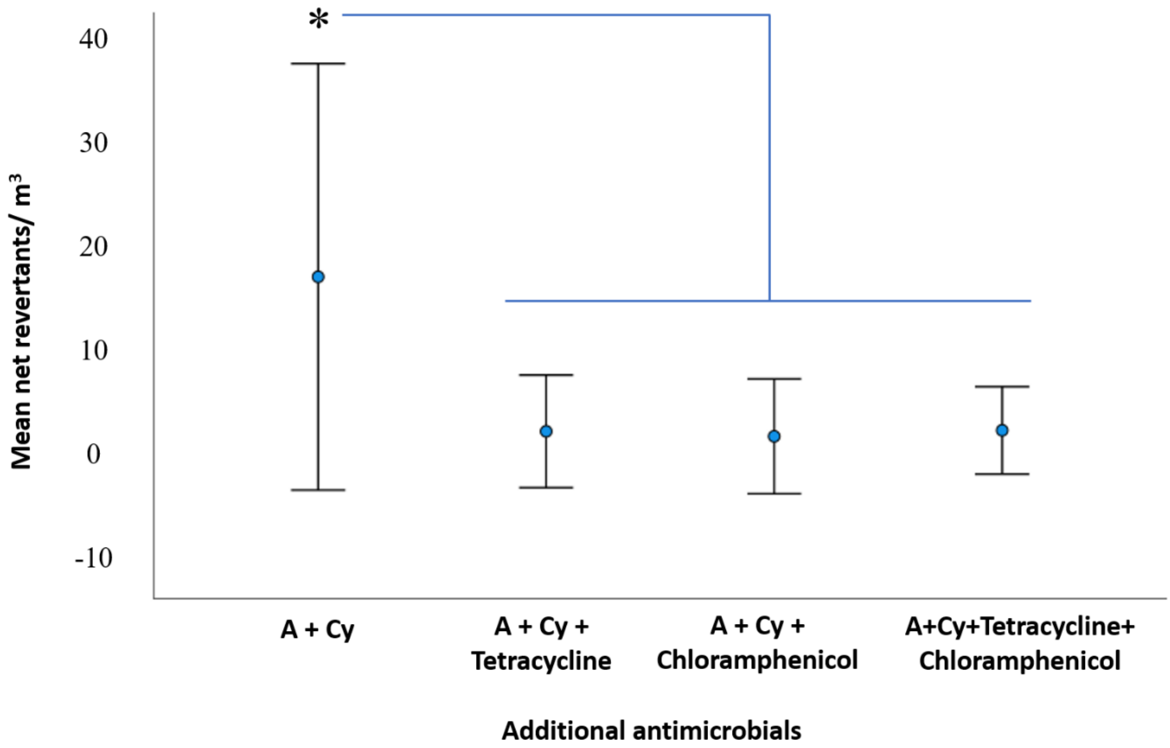
*
40
30
20
10
0
-10
Mean net revertants/ m3
A + Cy
A + Cy + Tetracycline
A + Cy + Chloramphenicol
A+Cy+Tetracycline+ Chloramphenicol
Additional antimicrobials
